# Circulating Metabolites Associated with Postprandial Satiety in Overweight/Obese Participants: The SATIN Study

**DOI:** 10.3390/nu13020549

**Published:** 2021-02-08

**Authors:** Lucía Camacho-Barcia, Jesús García-Gavilán, Christopher Papandreou, Thea T. Hansen, Jo A. Harrold, Graham Finlayson, John E. Blundell, Anders Sjödin, Jason C. G. Halford, Mònica Bulló

**Affiliations:** 1 Department of Biochemistry and Biotechnology, Faculty of Medicine and Health Sciences, University Rovira i Virgili (URV), 43201 Reus, Spain; lucamacho@gmail.com (L.C.-B.); jesusfrancisco.garcia@iispv.cat (J.G.-G.); 2 Institute of Health Pere Virgili—IISPV, University Hospital Sant Joan, 43201 Reus, Spain; 3 Consorcio CIBER, M.P. Fisiopatología de la Obesidad y Nutrición (CIBEROBN), Instituto de Salud Carlos III, 28029 Madrid, Spain; 4 Section for Obesity Research, Department of Nutrition, Exercise and Sports, University of Copenhagen, 2200 Copenhagen, Denmark; tha@nexs.ku.dk (T.T.H.); amsj@nexs.ku.dk (A.S.); 5 Department of Psychology, Institute of Population Health, University of Liverpool, Liverpool L69 3GL, UK; harrold@liverpool.ac.uk (J.A.H.); j.halford@leeds.ac.uk (J.C.G.H.); 6 Appetite Control & Energy Balance Research Group, School of Psychology, Faculty of Medicine & Health, University of Leeds, Leeds LS2 9JT, UK; g.s.finlayson@leeds.ac.uk (G.F.); j.e.blundell@leeds.ac.uk (J.E.B.)

**Keywords:** metabolomics, obesity, satiety

## Abstract

**Scope**: To identify a metabolomic profile related to postprandial satiety sensations involved in appetite control would help for a better understanding of the regulation of food intake. Methods and Results: A cross-sectional analysis of plasma metabolites was conducted over 151 overweight/obese adults from the “Satiety Innovation”-SATIN study, a randomized clinical trial of a 12-week weight-loss maintenance period. Postprandial satiety sensations (3 h-iAUC) were assessed by visual analogue scale (VAS) at the beginning and at the end of the study. Fasting plasma metabolites were profiled using a targeted multiplatform metabolomics approach before each appetite test meal. Associations between 124 metabolites and iAUC-satiety were assessed using elastic net linear regression analyses. The accuracy of the multimetabolite weighted models for iAUC-VAS was evaluated using a 10-fold cross-validation (CV) approach and the Pearson’s correlation coefficients were estimated. Five and three metabolites were selected in the first and the second assessments, respectively. Circulating glycine and linoleic acid concentrations were consistently and positively associated with higher iAUC-satiety in both visits. Sucrose and sphingomyelins (C32:2, C38:1) were negatively associated with iAUC-satiety in the first visit. The Pearson correlations coefficients between the metabolomic profiles and iAUC-satiety in the first and the second appetite assessments were 0.37 and 0.27, respectively. Conclusion: Higher glycine and linoleic acid were moderately but consistently associated with higher postprandial satiety in two different appetite assessments in overweight and obese subjects.

## 1. Introduction

Overweight and obesity are complex conditions developed from the interaction of several factors including physiologic, metabolic, cellular, and molecular processes but also cultural, socioeconomic, or behavioural aspects [1]. Strategies to control the increasing obesity pandemic are limited in number and efficacy. Bariatric surgery in extreme obesity is one of the most successful interventions, but with an associated risk of complications and mortality [2]. Pharmacology has also provided specific therapies targeting peripheral hormones and neural pathways to individuals with body mass index (BMI) over 30 kg/m^2^ for whom their condition compromised health. Several of these drugs were withdrawn due to unacceptable side effects [3]. However, because weight gain leading to obesity arises when energy intake exceeds energy expenditure over a prolonged period of time, to naturally limit calorie intake by targeting appetite sensations could be a successful strategy to promote weight management for the majority of people with overweight or obesity [4].

Impaired appetite control is considered an important contributor to weight gain and obesity development [5]. Evidence from clinical studies found a weaker satiety response to a test meal in obese subjects, reporting no relationship between their eating behaviour and appetite sensations [6]. These observations are not limited to obese subjects, and a “low satiety phenotype” was described [7]. Therefore, a better understanding of the complex biological processes involved in the control of appetite and food intake can contribute to a better management of body weight and obesity.

Extensive research efforts during the last decades have revealed much information of the neuroendocrine system and gut peptides related to satiety [3]. However, peripheral physiology and metabolic events also play an important regulatory role. Metabolomics afford profiling of hundreds of structurally and functionally diverse metabolites and provides an instantaneous snapshot of the physiological status of the organism. Metabolites function both as messengers by carrying data about the physiological environment, but also as transducers by directly controlling several physiological pathways. Although metabolomics have been extensively used in nutrition research, their use for the understanding of complex behaviours such as appetite is less explored. Thus, whereas the neuroendocrine pathways related to hunger and satiety have been widely studied and markers of appetite sensations described, metabolites related to the postprandial satiety period are unknown. Indeed, a recent review has claimed metabolomic profiling as a potentially powerful but relatively unexplored tool in the field of appetite [8]. Still, whether a specific metabolic profile can distinguish participants with higher postprandial satiety remains unknown. For this reason, we aimed to identify a metabolomic profile associated with postprandial satiety sensations after a test meal.

## 2. Material and Methods

### 2.1. Study Design and Subjects

The present analysis was nested in the European Commission Project Satiety Innovation (SATIN), a multicentre clinical trial aiming to evaluate the benefits of enhancing satiety on medium-term weight regain prevention. Eligible participants were women and men aged between 20 and 65 years with overweight/obesity (BMI 27–35 kg/m^2^) without comorbidities. Participants with weight changes previous to the study (±3 kg in the last three months), chronic medical conditions (type 1 or 2 diabetes, cardiovascular diseases, hypertension, chronic kidney diseases, liver diseases, active inflammatory bowel diseases, cancer, bariatric surgery, and other major surgical interventions, psychological or behavioural problems, psychiatric disorders), drug addictions, alcohol consumption above recommendations, and smoking (included smoking cessation within the last three months previous to start the study) were excluded from the study.

Briefly, after an initial 8-week low-calorie diet (LCD) period, participants achieving ≥8% of weight loss were included in the subsequent 12-week randomized double-blind parallel intervention phase for weight lost maintenance (WLM), with food products designed to reduce appetite or matching control products as placebo. Detailed information about study design, visits, and methods has been previously published [9].

The study and all procedures were conducted in accordance with the ethical principles set forth in the current version of the Declaration of Helsinki (Fortaleza, Brazil, October 2013) and the International Conference on Harmonization E6 Good Clinical Practice (ICH-GCP). The protocol was approved by the local institutional review boards and Ethics Committees of all the recruiting centres (15-07-30/7assN2 for the Spanish centre and H-15008553 for the Danish centre). Participants received written and oral information about the study procedures, and all participants provided written informed consent. All study procedures were aligned between sites before initiation of the study and on-site monitoring visits were carried out by an independent monitor. This trial was registered in clinicaltrials.gov (identifier: NCT02485743).

**Figure 1:**
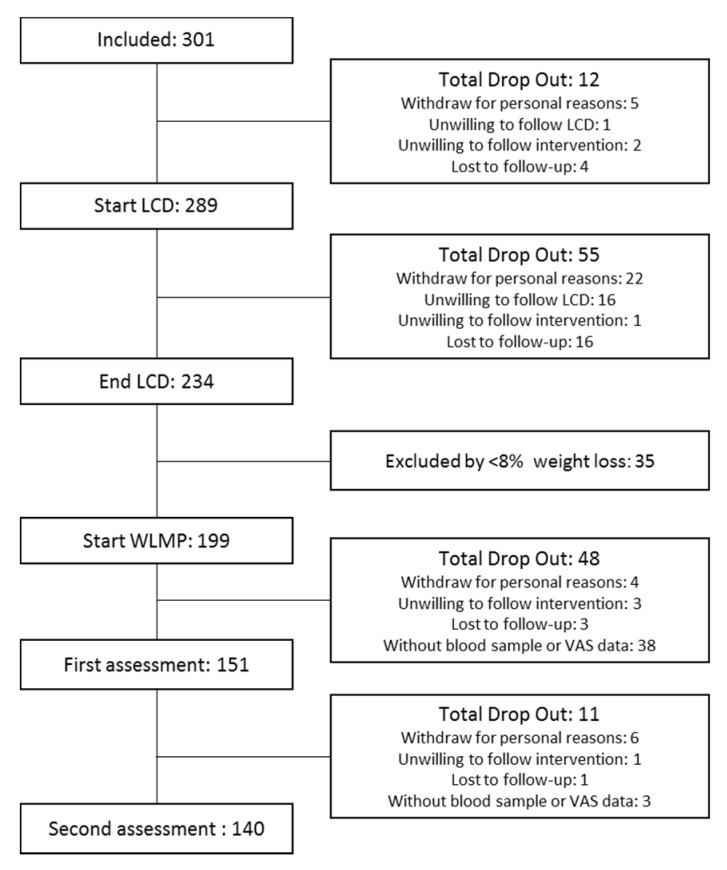
Flow-chart of the study.

From a total of 289 participants available at baseline, 151 participants achieving >8% weight loss during the initial 8 weeks and with available blood samples and visual analogue scale (VAS) data were considered in the current analysis (71 participants recruited in Reus and 80 in Copenhagen). For the second appetite assessment conducted after a 12-weeks of weight maintenance period, data of 140 participants (66 from Reus and 74 from Copenhagen) were available (Figure 1).

### 2.2. Assessment of Appetite

An 8 h appetite was assessed during study visits scheduled at the beginning (first visit) and after a 12-weeks (second visit) post maintenance period by a self-reported appetite evaluation, when subjects arrived to the centre facilities after an overnight fast and using the least strenuous means of transportation. For the appetite assessment, participants received a fixed breakfast meal (ham sandwich, coffee, milk, and orange juice), providing 478 Kcal corresponding to approximately 20% of the daily energy requirement for an average adult distributed as 55% of energy (E) as carbohydrates, 30%E as fat and 15%E as protein [10]. Self-reported appetite ratings were assessed using either electronic VAS (Lenovo^®^ thinkpad^®^ tablet 10; Evascale, build by Jakob Lund Laugesen, University of Copenhagen) (used in Copenhagen) or pen-and-paper VAS (used in Reus), a standard psychometric response scales of a 100-mm long horizontal line assessing feelings of satiety, fullness, hunger, desire to eat, and prospective food consumption [11,12]. For the present analysis, we considered appetite ratings pre- and immediately post-breakfast consumption (after overnight fasting) and hourly for a postprandial period of 3 h, completed in a controlled environment, with 0 indicating higher appetite/less satiety and 100 indicating lower appetite/more satiety [13,14].

### 2.3. Anthropometrical and Biochemical Measurements

All anthropometric measures were performed with the use of calibrated scales and by trained staff. Body weight was measured with participants wearing light clothes, height was measured on a wall-mounted stadiometer and BMI was then calculated. Waist circumference was measured midway between the lowest rib and the iliac crest using a nonelastic tape measure. Blood pressure was measured with participants in a resting position and using calibrated automatic devices (Omron HEM705IT, Reus, Spain; A&D Medical UA-779, Copenhagen, Denmark). Blood samples were collected in fasting conditions before starting each appetite assessments. Plasma glucose and lipid profile were measured using standard enzymatic automated methods (COBAS; Roche Diagnostics Ltd., Burgess Hill, United Kingdom).

### 2.4. Multiplatform Targeted Metabolomics

Metabolomic analysis was conducted in fasting blood samples using a multiplatform approach including gas and liquid chromatography coupled to high-resolution mass spectrometry (GC/LC-HRMS), and proton nuclear magnetic resonance (^1^H-NMR). The Bravo Automated Liquid Handling Platform from Agilent Technologies was used to extract metabolites from fasting plasma samples in 96-well format plates.

A protein precipitation extraction for GC-HRMS analysis was obtained by adding 400 of μL methanol: H_2_O (8:1) mixture to 100 μL of plasma. Supernatants were collected in new 96 well plates that contain internal standards. This plate was dryness with a vacuum centrifugation system (Speed Vac) and extracts were reconstituted with methoxyamine and incubated 90 min at 37 °C. Metabolites were sylilated with N-Methyl-N-(trimethylsilyl) trifluoroacetamide (MSTFA) + 1% trimethylchlorosilane (TMCS) at room temperature during 60 min. For LC-HRMS analysis and NMR analyses, lipidic fraction was obtained using a methanol/methyl-tert-butyl ether mixture. A small aliquot of the organic phase was dispensed and diluted 1:10 with methanol in 96-well plates containing deuterated internal standards for each family of lipids (Lipidomix SPLASH from Avanti Polar Lipid). The lipidomic analysis by NMR (^1^H-NMR) was conducted using a second aliquot of the organic phase dryness with Speed Vac. Extracts were reconstituted with a solution of CD_3_Cl: CD_3_OD with 4% D_2_O and 0.01% TMS (0.067 mM, Eretic Signal 6166 mM).

### 2.5. GC-HRMS Metabolomics Profiling

Samples were analysed in a 7890A Series gas chromatograph coupled to a 7000 GC-qTOF from Agilent Technologies using the J&W Scientific HP5-MS (30 m × 0.25 mm i.d., 0.25 µm film) chromatographic column and helium as carrier gas. Ionization was done by electronic impact (EI), recording data in Full Scan mode. Quantification was performed using the corresponding analytical standard for each determined metabolite and a deuterated internal standard depending on the family of metabolite. Internal standards used were succinic acid-d_4_, glycerol-^13^C_3_, norvaline, L-methionine-(carboxy-^13^C, methyl-d_3_), D-glucose-^13^C_6_, myristic-d_27_ acid, and alpha-tocopherol-d_6_.

### 2.6. LC-HRMS Lipidomics Profiling

The lipid species present in the plasma samples were determined by ultra-high performance chromatography coupled to quadrupole-time of flight high-resolution mass spectrometry 6550 (UHPLC-qTOF, Agilent Technologies). The ionization was performed in positive electrospray and mass calibration reference was used along all the analyses to maintain the mass accuracy below 5 ppm. Lipids were separated on a C18 reversed phase column (Kinetex C18-EVO, Phenomenex) and a ternary mobile phase (water/methanol/2-propanol) was used. The quantification of each lipid was made using one analytical standard and one deuterated internal standard for each lipid family (lysophosphatidylcholines, phosphatidylcholines, sphingomyelins, and triglycerides).

### 2.7. ^1^H-NMR Lipidomic Profiling

^1^H NMR spectra were recorded at 300K on an Avance III 600 spectrometer (Bruker^®^, Germany) operating at a proton frequency of 600.20 MHz using a 5 mm PBBO gradient probe. Lipidic samples were measured and recorded in procno 11 using a simple presaturation sequence (RD–90°–ACQ zgpr pulse program in Bruker^®^) to eliminate the residual water moisture of deuterated methanol. Solvent pre-saturation with irradiation power of 50 Hz was applied during recycling delay (RD = 5 s) and mixing time. The 90° pulse length was calibrated for each sample and varied from 10.42 to 11.49 ms. The spectral width was 12 kHz (20 ppm), and a total of 64 transients were collected into 64 k data points for each 1H spectrum. The exponential line broadening applied before Fourier transformation was of 0.3 Hz. The frequency domain spectra were manually phased and baseline-corrected using TopSpin software (version 2.1, Bruker). After pre-processing and visual checking of NMR dataset, specific 1H regions of diacylglycerols, triglycerides, and total lipids based on terminal methyl and methylene signals were identified in the spectra using a comparison into AMIX 3.9 software. Curated identified regions across the spectra were integrated using the same AMIX 3.9 software package and exported to an Excel spread sheet in order to give absolute concentrations. Information about the mass to charge ratio and retention time is shown in Supplementary Table S1.

### 2.8. Statistical Analysis

Baseline participants’ characteristics are described as means and standard deviations (SD) for quantitative variables, and percentages for categorical. Study participants’ characteristics were compared between the two appetite’s assessments using paired *t*-test for continuous variables and χ^2^ tests for categorical variables. Metabolites with equal or more than 20% of missing values were excluded. For those with less missing values, data were imputed using the random forest imputation method “missForest” function from the “randomForest” v 4.6-14 R package. A total of 9 metabolites were, therefore, excluded from the initial metabolomic panel (*n* = 133) and 124 metabolites remained into the analysis. Rank-based inverse normal transformation was applied to the metabolomics data to improve normality. Gaussian regression with an elastic net penalty was used to build a multimetabolite model for the incremental area under the curve (iAUC) of the VAS for satiety (“caret” v 6.0-84 and “glmnet” v 3.0-2 R package) for the first and second study visit. iAUC were calculated using the trapezoidal integration of pre- and immediately post-preload consumption, 1 h, 2 h, and 3 hours’ satiety ratings (trapz function of “pracma” v 2.2.9 R package). iAUC scores were adjusted for changes in body weight during the pre-visit weight-loss period before the first study visit and during the 12 week weight-maintenance period between the first and second study visit, using the residuals method [15]. A 10 cross-validation (CV) approach was performed, splitting the whole dataset into training and validation sets (80% and 20%, respectively). Subsequently, a 10-fold CV was performed within each training set to estimate the best value of the tuning parameter (lambda) that yielded the minimum mean-squared error (MSE). Models were optimized using argument best Tune of the “caret” R package. In order to report the coefficients from each CV iteration, s = “lambda.min” was selected, as it gives the minimum mean CV error. The alpha parameter was also estimated using 0.1 increments from 0 (i.e., Ridge regression) to 1 (i.e., Lasso regression). The alpha value of the model with the best predicting accuracy in the validation sets was 0.8. Weighted models for each training-validation datasets (i.e., for every 80–20 split datasets) were constructed using the metabolite coefficients obtained from the elastic net regression of each training set. Pearson correlation coefficients (95% confidence interval (CI)) were computed to assess the performance of the multimetabolite models for iAUC satiety in the validation set. Pearson correlation coefficients (95% confidence interval (CI)) were computed to assess the performance of the multimetabolite models for iAUC satiety in the validation set. This approach was also used to correlate the identified multimetabolite model with fasted VAS and the amount of energy intake at lunch and dinner in the day of the study. For reproducibility purposes, the regression coefficients were presented using 10 iterations of the 10-CV elastic regression approaches in the whole dataset. Pearson’s rank correlation analysis and scatter plots for the association between each selected metabolite and the VAS scores were conducted. All the analyses were performed using R statistical software (v 3.6.1) (R Foundation for Statistical Computing, Vienna, Austria)

## 3. Results

The general characteristics of the 151 and 140 participants in the first and appetite assessments are shown in Table 1. The mean age was 47.5 ± 10 years, with a BMI of 27.5 ± 2.0 kg/m^2^ and 27.7 ± 2.2 kg/m2 for the first and second appetite assessment, respectively. The mean iAUC-VAS was 1056.5 ± 290.1 and 1082.8 ± 285.1, respectively. Total, High-density lipoprotein (HDL) and Low-density lipoprotein (LDL) cholesterol were significantly higher in subjects in the second appetite assessment. The current metabolomics platforms identified different metabolites, of which triglyceride species 52.3 and 52.2 had the highest concentrations, while sucrose and docosahexaenoic had the lowest concentrations (Table S1).

**Figure 2:**
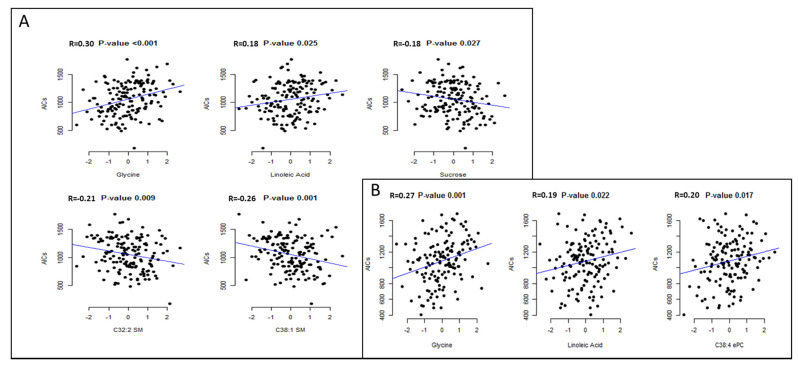
Scatter plots of the data between the metabolite levels and the VAS scores at the first (**A**) and the second (**B**) appetite assessment. Pearson correlation coefficients for the association of each metabolite selected in the elastic net regression with the VAS scores.

Table 2 displays the mean coefficient values and SDs for those metabolites selected 10 times in the 10 CV analyses for the two study visits. In the first study visit analysis, both glycine and linoleic acid were positively associated with higher iAUC satiety, whereas sucrose, and two sphingomyelin (SM) species (C32:2 and C38.1) were inversely associated with increased satiety sensations. Consistently, both glycine and linoleic acid were positively associated with higher iAUC satiety in the second study visit analysis. Higher iAUC satiety was also positively associated with C38:4e phosphatidylcholine but only in the second study visit. In the training set, the unbiased metabolomic profiles acquired using the 10-fold cross-validation approach were significantly correlated with iAUC satiety in both study visits (*r* = 0.37 and *r* = 0.27, respectively) (Table 3). Similar results were obtained for fasted VAS (*r* = 0.22 (0.07, 0.37), *p* = 0.006). Scatter plots of the metabolites selected by elastic net and the VAS scores at the first and the second appetite assessment are shown in Figure 2.

## 4. Discussion

In the present study, circulating glycine and linoleic acid concentrations were consistently and positively associated with higher postprandial satiety sensations in two different appetite assessments in overweight/obese participants of the SATIN study.

The duration and the intensity of postprandial satiety result from a complex interaction between internal and external factors including physiology, cognitive and sensory factors, as well as intrinsic characteristics of the meal (i.e., energy and nutrient content) [15]. A complex cascade of hormonal and other molecules are secreted to be integrated in the brain, which influence the satiety response [16]. However, individual differences result in variability in the processing of satiety that can lead to overeating due to a diminished sensitivity to satiety signals and increased susceptibility to the food environment and its obesogenic effects [17]. It has been proposed that some of these individual differences in satiety responsiveness have a genetic basis [17] but other causal factors can influence and become biomarkers of appetite [18].

Our results showed a significant relationship between higher circulating glycine concentrations with a higher satiety response. Glycine is present in the nervous system and acts as a neurotransmitter binding to several receptors and inhibiting many chemical processes [19]. Its effects seem to be similar to the gamma-Aminobutyric acid (GABA) action. An increased glycine function effect may result as a similar increment in the GABAergic neurotransmission [19]. It has been reported that low plasma concentrations of glycine are associated with obesity and T2D and that in the long-term its deficiency may be related to the origin of other metabolic disorders like insulin resistance and nonalcoholic fatty liver disease [20]. The association between plasma concentrations of amino acids and satiety has been previously assessed in a post Roux-en-Y gastric bypass surgery sample [21]. According to this study, participants with greater postprandial satiety responses had a significantly higher median iAUC of several plasma amino acids concentrations, including glycine. The ‘protein-stat theory’ proposed by Millward suggested that amino acids sensors could potentially provide a mechanism in which the energy demands originating from fat-free mass are translated into food intake [21].

Higher circulating concentrations of linoleic acid were also positively associated with higher postprandial satiety. To our knowledge, there is no previous study on circulating individual fatty acids relationships with appetite. Reduced appetite ratings were observed acutely after administering emulsions enriched with linoleic acid compared with oleic and stearic acid through upper intestinal perfusions to lean human subjects [22]. A previous randomized, crossover study of healthy subjects concluded that when infused into the ileum, triacylglycerol with unsaturated fatty acids including linoleic acid increase satiety compared with triacylglycerol with saturated fatty acids [23]. Total levels of free fatty acids have been previously associated with appetite control [8], however, no indication of a circulating individual fatty acids effect on appetite has been established yet. Results from a study that aimed to identify a potential effect of pharmacological modifications of fatty acid metabolism on human satiety showed that an inhibitor of lipogenesis increased the intensity and duration of satiety. It was hypothesized that this effect may be produced through the increment of non-esterified fatty acids, increasing their disposal for oxidation [24].

Although not consistently found through the appetite assessments, the SMs C32:1 and C38:2 were inversely associated with satiety sensations in the first assessments. A reduction in the sphingolipid-1-phosphate to C14:1 ceramide ratio was correlated with a reduction in postprandial satiety via the neuropeptide Y-like receptor (NepYr) in Drosophila, supporting the role of ceramides in appetite regulation and caloric-intake dependent obesity [25]. Since SMs are synthesized from ceramides, lower SM levels could be interpreted as lower ceramide concentrations that are inversely associated with increased satiety. Whether SM could modulate satiety via the NepYr deserves further investigation [25].

Although there are small amounts of sucrose in blood as it is mainly hydrolyzed and absorbed as glucose and fructose, in our study, plasma sucrose levels were associated with lower postprandial satiety on the first study visit. The effect of sucrose and other types of sugars on the appetite mechanisms is still not fully understood. In short test meals, sucrose intake produces an immediate insulin secretion, producing appetite regulation signals that contribute to satiety and the suppression of appetite and food intake [26]. However, the long-term effect is still widely debated due to the vast body of data, which inversely associate sucrose consumption with body mass index and the risk of obesity [27]. A previous study performed in animal models with the objective of recognising the effects of sucrose and other types of sugars on peripheral and central appetite showed that after 24 h of drinking sucrose, total serum peptide YY (PYY), a peripheral appetite signal [28], was significantly increased compared to a water control [29]. Yet, after two weeks consuming sucrose solutions, the serum PYY concentration was significantly lower that the water control group. Results also showed an increased in the caloric intake and in the body weight gain in this group, as an up-regulation of the hypothalamic CB1 mRNA, being CB1 an endocannabinoid receptor involved in feeding reward [30]. On the other hand, they also observed an increase of serum levels of leptin, an anorexigenic hormone [31], and a down-regulation of hypothalamic NPY mRNA, a hunger neuropeptide [32]. Authors later hypothesized that the caloric over-consumption and weight gain produced by the sucrose consumption may be associated to the activation of hunger signals and reward components, as well as the decline of satiety signals. Additionally, the increment in serum leptin and the down-regulation of NPY mRNA may be a possible defence mechanism against the weight-promoting effect of the sugar solutions. Interestingly, the identified metabolomic profile was not significantly correlated with energy intake at lunch and dinner. We also found no correlation between VAS and energy intake, which is in line with previous studies suggesting that self-reported appetite ratings do not reliably predict energy intake [33]. The feeling of appetite may affect energy intake but also be a consequence of the amount and type of food/meal eaten. Therefore, the metabolites identified for VAS do not seem to be related to the amount of energy ingested.

Our study has limitations that need to be acknowledged. First, even though VAS are widely used to assess the physiological and psychological dimensions of appetite sensations, and provide reproducible and valid information, they were self-report and, therefore, subject to some degree of measurement error [12]. For this reason, we assessed the relationship between metabolites and VAS on two separate occasions. Secondly, we evaluated a sample of individuals with overweight and obesity that could limit the generalizability of our results to other populations. Thirdly, due to the cross-sectional design, causation and direction of causality cannot be inferred; therefore, currently both directions are plausible and require further investigation. Finally, the lack of reproducibility in four of the selected metabolites could be due to a smaller sample size in the second appetite assessment or due to other confounders beyond those considered in our study. On this regard, it is important to highlight that no significant differences were observed in anthropometrical and nutritional measurements between the two assessments, while breakfast’s food and nutritional composition was equal between the assessments.

In conclusion, the present study identified a small set of metabolites moderately associated with postprandial satiety in a study population with overweight/obesity. These findings could help us to better understand the metabolic pathways implicated in satiety and in the future may result in a helpful tool for the design of more effective strategies for appetite control.

## Figures and Tables

**Table 1:** Characteristics of the study participants.

Characteristics	First Appetite Assessment (*n* = 151)	Second Appetite Assessment (*n* = 140)	*p*-Value
Age, years	48 ± 10	48 ± 10	0.594
Sex, women	74 (117)	75 (105)	0.821
Weight, kg	78.4 ± 9.3	78.9 ± 9.8	0.495
BMI, kg/m^2^	27.5 ± 2.0	27.7 ± 2.2	0.383
Waist circumference, cm	90.8 ± 8.3	91.4 ± 8.8	0.425
SBP, mm Hg	121 ± 14	121 ± 14	0.820
DBP, mm Hg	77 ± 9	77 ± 9	0.839
Glucose, mg/dL	91.8 ± 8.7	91.0 ± 8.3	0.439
Total cholesterol, mg/dL	177.5 ± 31.7	194.9 ± 37.2	<0.001
HDL-C, mg/dL	51.0 ± 12.7	61.4 ± 15.5	<0.001
LDL-C, mg/dL	108.5 ± 27.2	116.2 ± 32.0	0.029
Triglycerides, mg/dL	90.1 ± 36.0	85.6 ± 41.0	0.324
Total energy intake, kcal/day	1523.27 ± 572.05	1552.60 ± 562.28	0.658
Carbohydrates intake (%E)	40.03±7.25	40.35±7.00	0.704
Protein intake (%E)	21.44±5.19	19.89±4.16	0.006
Fat intake (%E)	35.63±7.66	36.07±6.62	0.609
Fiber, g/day	17.89±8.04	18.15±7.98	0.784
iAUC-VAS	1056.5 ± 290.1	1082.8 ± 285.1	0.769

Mean ± SD or % (N). Abbreviations: BMI, body mass index; SBP, systolic blood pressure; DBP, diastolic blood pressure; HDL-C, high-density lipoprotein cholesterol; LDL-C, low-density lipoprotein; iAUC-VAS, integrated area under the curve of the visual analogic scale of satiety.

**Table 2:** List of coefficients of metabolites selected 10 times in the ten times iterated 10-fold-cross validation of the elastic continuous regression analyses in the whole Satiety Innovation (SATIN) dataset and including the two satiety assessments.

Metabolites	Elastic Gaussian Regression (First Assessment)	Elastic Gaussian Regression (Second Assessment)
	Mean ± SD	Mean ± SD
Glycine	41.70 ± 7.32	31.83 ± 3.30
Linoleic Acid	5.80 ± 3.51	9.20 ± 3.17
Sucrose	−7.71 ± 6.11	-
C32.2 SM	−9.91 ± 4.22	-
C38.1 SM	−19.92 ± 2.34	-
C38.4 ePC	-	7.11 ± 2.90

Data are shown as mean of elastic net regression coefficient ± standard deviation (SD). SM, Sphingomyelin; PC, phosphatidylcholine.

**Table 3:** Pearson correlation coefficients for the iAUC-VAS corresponding to the two satiety assessments.

Appetite Assessment	Coefficients (95% CI)	*p*-Values	Total Metabolites with Positive Coefficients	Total Metabolites with Negative Coefficients
First assessment	0.37 (0.23, 0.51)	<0.001	2	3
Second assessment	0.27 (0.11, 0.42)	0.001	3	-

All metabolites were selected 10 times in the cross-validation procedure for the elastic net Gaussian regression using “lambda.min” option. Abbreviations: 95%CI, 95% confidence interval.

## Data Availability

Data generated and analysed in the framework of the SATIN Consortium are not publicly available due to national data regulations and for ethical reasons, because study participants only gave their consent for the use of their data by the SATIN Consortium investigators. However, collaboration for data analyses can be requested by sending a letter to the corresponding author. The request would be evaluated by the SATIN Steering Committee for deliberation.

## Supplementary Materials

The following are available online at https://www.mdpi.com/2072-6643/13/2/549/s1. Supplementary Table S1. List of analysed metabolites.
