# Supplementary material for: Circulating Metabolites Associated with Postprandial Satiety in Overweight/Obese Participants: The SATIN Study
Source: Nutrients. 2021 Feb 8;13(2):549. doi: 10.3390/nu13020549 (PMC7916078; doi:10.3390/nu13020549)
Supplement: Supplementary file 1 [file nutrients-13-00549-s001.pdf]

Supplemental Table 1. List of analysed metabolites

						First appetite assessment			Second appetite assessment		
**names**	**name**	**Plattform**	**Quantitative ion (m/z)**	**Retention Time (min)**	**Chemical Shift (ppm)**	**mean**	**median**	**SD**	**mean**	**median**	**SD**
2.HbutA	2-Hydroxybutanoic acid in plasma	GC-MS	131,0895	6,9	–	0,180	0,173	0,060	0,178	0,161	0,060
3.HbutA	3-Hydroxybutanoic acid in plasma	GC-MS	117,0731	7,3	–	2,083	1,033	4,064	1,098	0,564	1,257
Ala	Alanine in plasma	GC-MS	116,0916	6,5	–	2,076	2,071	0,623	2,188	2,138	0,691
alphaToco	alpha-Tocopherol in plasma	GC-MS	237,1337	26,3	–	0,073	0,069	0,020	0,075	0,073	0,020
Choles	Cholesterol in plasma	GC-MS	129,0746	26,4	–	3,198	3,172	0,517	3,169	3,148	0,499
CitA	Citric acid in plasma	GC-MS	273,1050	15,7	–	0,960	0,919	0,263	0,854	0,808	0,225
Fruc	Fructose in plasma	GC-MS	307,1650	16,4	–	0,239	0,228	0,043	0,243	0,234	0,045
GliA	Gliceric acid in plasma	GC-MS	189,0782	9,8	–	0,080	0,077	0,016	0,070	0,070	0,007
Glu	Glucose in plasma	GC-MS	319,1592	16,6	–	13,185	13,169	1,325	13,131	13,023	1,317
GlutA	Glutamic acid in plasma	GC-MS	246,1392	13,4	–	0,366	0,329	0,188	0,309	0,270	0,149
GlyA	Glycolic acid in plasma	GC-MS	177,0764	6,0	–	0,072	0,071	0,009	0,073	0,072	0,012
Glycerol	Glycerol in plasma	GC-MS	218,1185	9,0	–	0,353	0,324	0,134	0,361	0,308	0,210
Glycine	Glycine in plasma	GC-MS	174,1153	9,4	–	1,629	1,558	0,509	1,538	1,432	0,504
Isoleu	Isoleucine in plasma	GC-MS	158,1404	9,2	–	0,210	0,197	0,061	0,211	0,202	0,055
LA	Lactic acid in plasma	GC-MS	190,0880	5,8	–	4,152	3,762	1,592	4,682	3,999	2,785
Leu	Leucine in plasma	GC-MS	158,1432	8,9	–	0,325	0,310	0,084	0,337	0,327	0,078
LinoA	Linoleic acid in plasma	GC-MS	117,0382	19,2	–	0,307	0,276	0,155	0,284	0,264	0,145
Lys	Lysine in plasma	GC-MS	200,1102	16,0	–	0,900	0,889	0,268	0,976	0,970	0,268
Meth	Methionine in plasma	GC-MS	128,0909	12,2	–	0,134	0,134	0,024	0,142	0,140	0,025
OleicA	Oleic acid in plasma	GC-MS	117,0382	19,3	–	2,041	1,872	0,982	1,785	1,530	0,924
Orn	Ornithine in plasma	GC-MS	142,1063	15,6	–	0,282	0,253	0,106	0,315	0,290	0,123
PalA	Palmitic acid in plasma	GC-MS	117,0382	17,7	–	0,811	0,765	0,303	0,755	0,685	0,315
Phe	Phenylalanine in plasma	GC-MS	218,1050	13,5	–	0,357	0,350	0,054	0,377	0,370	0,058
Proline	Proline in plasma	GC-MS	142,1082	9,3	–	0,361	0,331	0,128	0,350	0,333	0,112
Ser	Serine in plasma	GC-MS	204,1316	10,2	–	0,188	0,181	0,041	0,178	0,174	0,037
StearicA	Stearic acid in plasma	GC-MS	117,0382	19,5	–	1,145	1,053	0,403	1,099	0,972	0,408
Sucrose	Sucrose in plasma	GC-MS	361,1790	23,2	–	0,004	0,003	0,003	0,004	0,003	0,002
Thr	Threonine in plasma	GC-MS	218,1053	10,6	–	0,283	0,277	0,068	0,283	0,280	0,073
Tryp	Tryptophan in plasma	GC-MS	202,1134	19,5	–	2,417	2,270	0,675	2,620	2,489	0,713

Tyr	Tyrosine in plasma	GC-MS	179,0902	16,9	-	0,650	0,651	0,146	0,702	0,676	0,155
Val	Valine in plasma	GC-MS	144,1229	8,1	-	0,933	0,910	0,199	0,957	0,958	0,180
LPC 14.0	Lysophosphatidylcholine 14:0 in plasma	LC-MS	468.3085	1,3	-	821,468	818,278	10,206	823,379	821,516	9,866
LPC 15.0	Lysophosphatidylcholine 15:0 in plasma	LC-MS	482.3241	1,5	-	797,584	797,302	4,436	797,117	796,754	4,865
LPC 16.0	Lysophosphatidylcholine 16:0 in plasma	LC-MS	496.3398	1,8	-	2791,603	2743,942	469,895	2589,960	2550,417	495,012
LPC 16.0.e	Lysophosphatidylcholine 16:0 e in plasma	LC-MS	482.3605	1,9	-	800,071	799,875	4,682	797,946	797,489	5,055
LPC 16.1	Lysophosphatidylcholine 16:1 in plasma	LC-MS	494.3241	1,5	-	800,181	798,327	14,825	799,078	798,069	14,181
LPC 16.1.e	Lysophosphatidylcholine 16:1 e in plasma	LC-MS	480.3449	2,0	-	802,732	802,214	5,094	800,311	799,956	5,144
LPC 17.0	Lysophosphatidylcholine 17:0 in plasma	LC-MS	510.3554	2,0	-	765,508	764,367	8,745	763,290	763,646	9,507
LPC 18.0	Lysophosphatidylcholine 18:0 in plasma	LC-MS	524.3711	2,2	-	1216,739	1204,303	157,413	1183,041	1181,335	168,632
LPC 18.0.e	Lysophosphatidylcholine 18:0 e in plasma	LC-MS	510.3918	2,5	-	745,018	744,900	1,338	744,604	744,500	1,376
LPC 18.1	Lysophosphatidylcholine 18:1 in plasma	LC-MS	522.3554	1,9	-	1190,588	1177,205	133,313	1167,633	1170,263	145,152
LPC 18.2	Lysophosphatidylcholine 18:2 in plasma	LC-MS	520.3398	1,6	-	1267,857	1211,921	234,191	1245,057	1224,490	204,593
LPC 20.0	Lysophosphatidylcholine 20:0 in plasma	LC-MS	552.4024	2,7	-	731,848	731,830	0,932	731,746	731,661	1,070
LPC 20.1	Lysophosphatidylcholine 20:1 in plasma	LC-MS	550.3867	2,3	-	692,102	691,827	2,222	691,042	691,008	2,228
LPC 20.3	Lysophosphatidylcholine 20:3 in plasma	LC-MS	546.3554	1,8	-	723,187	721,307	13,741	727,914	725,204	15,708
LPC 20.4	Lysophosphatidylcholine 20:4 in plasma	LC-MS	544.3398	1,7	-	781,012	778,655	36,085	773,678	766,796	35,862
LPC 22.6	Lysophosphatidylcholine 22:6 in plasma	LC-MS	568.3398	1,7	-	688,196	686,658	11,151	685,899	685,332	11,110
PC 30.0	Phosphatidylcholine 30:0 in plasma	LC-MS	706.5381	4,1	-	1100,891	1077,207	94,919	1138,819	1121,008	91,045
PC 32.0	Phosphatidylcholine 32:0 in plasma	LC-MS	734.5694	4,7	-	2616,340	2567,357	312,803	2695,284	2636,100	345,626
PC 32.1	Phosphatidylcholine 32:1 in plasma	LC-MS	732.5538	4,2	-	1834,105	1720,317	467,638	2017,125	1922,429	516,673
PC 32.1.e	Phosphatidylcholine 32:1 e in plasma	LC-MS	718.5745	4,7	-	1018,644	1015,206	27,990	1018,003	1016,097	28,753
PC 32.2	Phosphatidylcholine 32:2 in plasma	LC-MS	730.5381	3,9	-	932,191	918,014	61,157	960,633	952,577	58,550
PC 33.1	Phosphatidylcholine 33:1 in plasma	LC-MS	746.5694	4,5	-	945,461	939,172	41,282	965,858	962,223	46,063
PC 34.0	Phosphatidylcholine 34:0 in plasma	LC-MS	762.6008	5,5	-	1114,139	1114,696	53,823	1126,227	1126,686	67,155
PC 34.1.e	Phosphatidylcholine 34:1 e in plasma	LC-MS	746.6058	5,4	-	1281,761	1274,070	71,982	1294,093	1283,555	83,296
PC 34.2	Phosphatidylcholine 34:2 in plasma	LC-MS	758.5694	4,5	-	23183,934	21432,028	12902,655	27055,850	26468,689	13214,289
PC 34.2.e	Phosphatidylcholine 34:2 e in plasma	LC-MS	744,5918	5,1	-	940,240	934,478	32,540	943,346	935,697	38,727
PC 34.3.e	Phosphatidylcholine 34:3 e in plasma	LC-MS	742,5778	4,8	-	1804,038	1777,816	199,404	1822,707	1802,199	213,288

PC 34.4	Phosphatidylcholine 34:4 in plasma	LC-MS	754.5381	4,0	-	888,231	883,521	30,770	903,626	897,132	32,468
PC 35.1	Phosphatidylcholine 35:1 in plasma	LC-MS	774.6007	5,3	-	895,950	894,916	33,721	906,258	901,964	41,984
PC 35.2	Phosphatidylcholine 35:2 in plasma	LC-MS	772,5851	5,0	-	1905,699	1881,201	227,054	1944,451	1939,009	205,681
PC 36.1	Phosphatidylcholine 36:1 in plasma	LC-MS	788.6164	5,8	-	5761,056	5443,111	1356,576	6469,075	6242,781	1630,770
PC 36.2.e	Phosphatidylcholine 36:2 e in plasma	LC-MS	772.6215	5,1	-	954,421	952,981	20,622	956,257	954,020	22,802
PC 36.3	Phosphatidylcholine 36:3 in plasma	LC-MS	784.5851	4,8	-	15787,309	15993,293	3788,687	16845,387	17363,591	4379,147
PC 36.4.e	Phosphatidylcholine 36:4 e in plasma	LC-MS	768.5902	5,0	-	2593,568	2550,547	320,179	2736,112	2597,451	503,290
PC 36.5	Phosphatidylcholine 36:5 in plasma	LC-MS	780.5538	4,3	-	2613,806	2186,603	1518,635	2817,432	2567,291	1156,578
PC 36.5.e	Phosphatidylcholine 36:5 e in plasma	LC-MS	766.5762	4,7	-	2226,934	2196,960	255,924	2289,362	2227,407	321,145
PC 37.4	Phosphatidylcholine 37:4 in plasma	LC-MS	796,5851	5,0	-	1146,222	1141,997	60,098	1151,075	1140,148	73,648
PC 38.3	Phosphatidylcholine 38:3 in plasma	LC-MS	812.6164	5,6	-	5049,141	4801,450	1509,577	5966,246	5675,049	1713,844
PC 38.4	Phosphatidylcholine 38:4 in plasma	LC-MS	810.6007	5,4	-	9553,268	9436,454	1915,309	10008,824	10089,222	2142,753
PC 38.4.e	Phosphatidylcholine 38:4 e in plasma	LC-MS	796.6215	5,9	-	1350,487	1336,139	95,563	1398,219	1388,717	114,757
PC 38.5	Phosphatidylcholine 38:5 in plasma	LC-MS	808.5851	5,0	-	4797,452	4741,783	844,001	5046,710	4984,235	992,259
PC 38.5.e	Phosphatidylcholine 38:5 e in plasma	LC-MS	794.6058	5,2	-	868,357	864,258	38,576	868,048	864,338	42,730
PC 38.6	Phosphatidylcholine 38:6 in plasma	LC-MS	806.5694	4,4	-	9092,859	9131,301	1829,400	8920,868	9021,717	2061,715
PC 40.4	Phosphatidylcholine 40:4 in plasma	LC-MS	838.6320	6,0	-	897,053	888,619	37,510	908,839	904,875	42,082
PC 40.4.e	Phosphatidylcholine 40:4 e in plasma	LC-MS	824.6528	6,4	-	804,759	802,893	10,434	807,602	807,280	10,839
PC 40.5.e	Phosphatidylcholine 40:5 e in plasma	LC-MS	822.6371	5,9	-	834,675	831,247	18,367	835,606	833,967	17,871
PC 40.6	Phosphatidylcholine 40:6 in plasma	LC-MS	834.6007	5,4	-	2565,844	2533,631	534,469	2666,819	2606,072	684,969
PC 42.5.e	Phosphatidylcholine 42:5 e in plasma	LC-MS	850.6684	6,4	-	779,952	776,879	13,525	779,391	778,769	11,510
PE 36.5.e	Phosphatidylethanolamine 36:5 e in plasma	LC-MS	724.5276	4,4	-	539,447	535,627	71,947	572,658	560,036	81,102
PE 38.5.e	Phosphatidylethanolamine 38:5 e in plasma	LC-MS	752.5589	5,5	-	632,448	620,612	94,887	684,741	650,638	127,121
PE 38.6.e	Phosphatidylethanolamine 38:6 e in plasma	LC-MS	750,5449	5,2	-	739,653	732,758	91,392	785,226	769,036	119,123
SM 32.1	Sphingomyelin 32:1 in plasma	LC-MS	675.5436	3,5	-	1807,415	1785,585	172,659	1915,869	1876,749	227,073
SM 32.2	Sphingomyelin 32:2 in plasma	LC-MS	673.5279	3,3	-	1192,688	1192,377	8,705	1197,172	1196,232	10,487
SM 33.1	Sphingomyelin 33:1 in plasma	LC-MS	689.5592	3,7	-	1288,815	1285,630	46,536	1301,212	1296,838	52,024
SM 34.1	Sphingomyelin 34:1 in plasma	LC-MS	703.5749	4,0	-	3968,712	3933,642	582,751	4063,763	4003,278	652,740
SM 34.2	Sphingomyelin 34:2 in plasma	LC-MS	701.5592	3,7	-	1970,957	1951,765	159,325	1962,544	1940,559	186,418
SM 35.1	Sphingomyelin 35:1 in plasma	LC-MS	717,5905	4,4	-	1157,896	1155,155	20,056	1162,429	1160,927	22,493
SM 36.0	Sphingomyelin 36:0 in plasma	LC-MS	733.6218	4,8	-	1129,073	1124,396	20,840	1125,730	1123,720	18,045
SM 36.1	Sphingomyelin 36:1 in plasma	LC-MS	731.6062	4,6	-	1566,633	1559,898	148,202	1567,698	1550,146	160,655

SM 36.2	Sphingomyelin 36:2 in plasma	LC-MS	729.5905	4,2	-	1605,601	1597,038	129,221	1570,040	1562,611	120,515
SM 38.1	Sphingomyelin 38:1 in plasma	LC-MS	759.6375	5,4	-	1452,611	1452,335	137,300	1547,578	1542,141	156,684
SM 38.2	Sphingomyelin 38:2 in plasma	LC-MS	757.6218	4,8	-	1357,899	1346,829	57,211	1359,414	1353,408	67,186
SM 40.1	Sphingomyelin 40:1 in plasma	LC-MS	787.6688	6,2	-	1599,306	1585,331	160,740	1705,965	1664,086	197,894
SM 40.2	Sphingomyelin 40:2 in plasma	LC-MS	785.6531	5,7	-	1766,654	1766,486	138,538	1816,236	1799,136	172,091
SM 41.1	Sphingomyelin 41:1 in plasma	LC-MS	801.6844	6,3	-	1435,828	1422,258	101,497	1556,114	1538,766	131,013
SM 41.2	Sphingomyelin 41:2 in plasma	LC-MS	799.6688	6,1	-	1028,623	1026,180	27,378	1053,393	1049,533	35,627
SM 42.1	Sphingomyelin 42:1 in plasma	LC-MS	815.7001	6,5	-	1191,650	1183,741	90,816	1262,382	1234,106	122,625
SM 42.2	Sphingomyelin 42:2 in plasma	LC-MS	813.6844	6,2	-	3068,119	3001,962	490,672	2999,405	2962,941	524,841
SM 42.3	Sphingomyelin 42:3 in plasma	LC-MS	811.6688	5,8	-	2468,025	2427,324	322,094	2401,153	2346,456	336,229
TG 50.1	Triacylglycerides 50:1 in plasma	LC-MS	850.7858	9,7	-	34432,404	18773,453	38391,195	43080,786	22292,381	66047,027
TG 50.2	Triacylglycerides 50:2 in plasma	LC-MS	848.7702	9,3	-	52506,862	38950,341	40992,327	54565,070	42051,176	56663,697
TG 50.3	Triacylglycerides 50:3 in plasma	LC-MS	846.7545	8,9	-	15677,993	10380,924	14932,732	15979,254	10648,864	20559,583
TG 52.2	Triacylglycerides 52:2 in plasma	LC-MS	876.8015	9,8	-	170943,344	150215,063	92980,566	159399,269	131076,991	105834,640
TG 52.3	Triacylglycerides 52:3 in plasma	LC-MS	874.7858	9,4	-	185668,464	160988,803	106402,349	161625,078	143191,179	98893,206
TG 52.4	Triacylglycerides 52:4 in plasma	LC-MS	872.7702	9,1	-	51973,847	43254,839	43465,987	43279,217	32722,882	39150,808
TG 54.2	Triacylglycerides 54:2 in plasma	LC-MS	904.8328	10,3	-	13229,521	10497,744	14548,541	11687,135	9203,131	12628,658
TG 54.3	Triacylglycerides 54:3 in plasma	LC-MS	902.8171	10,0	-	46994,162	36821,959	37271,781	37338,873	30366,905	28566,590
TG 54.4	Triacylglycerides 54:4 in plasma	LC-MS	900.8015	9,6	-	52869,636	38868,672	48061,124	40194,734	33803,133	30466,163
TG 54.5	Triacylglycerides 54:5 in plasma	LC-MS	898.7858	9,2	-	34102,120	18627,155	52012,327	23711,000	14566,408	28630,322
ARA.EPA	Arachidonic acid + Eicosapentaenoic acid in serum	NMR	-	-	2,13	0,006	0,006	0,001	0,006	0,006	0,001
DHA	Docosahexaenoic acid in serum	NMR	-	-	2,41	0,002	0,002	0,000	0,002	0,002	0,000
Echr	Esterified Cholesterol in serum	NMR	-	-	1,04	3,489	3,376	0,737	3,783	3,717	0,918
FAC	Fatty acyl chains in serum	NMR	-	-	0,89	105,648	104,521	18,980	109,667	106,841	22,524
FChr	Free Cholesterol in serum	NMR	-	-	1,01	1,700	1,671	0,332	1,818	1,791	0,395
LCh	Lysophosphatidyl Choline in serum	NMR	-	-	3,23	1,862	1,831	0,349	2,009	1,999	0,416
LINOLEIC	LINOLEIC in serum	NMR	-	-	2,78	0,026	0,026	0,003	0,026	0,026	0,003
MUFA	monounsaturated fatty acid in serum	NMR	-	-	5,38	0,074	0,075	0,007	0,075	0,076	0,008
PhCh	Phosphatidyl Choline in serum	NMR	-	-	3,21	2,346	2,301	0,429	2,510	2,479	0,529
PUFA	polyunsaturated fatty acid in serum	NMR	-	-	2,85	0,048	0,048	0,006	0,047	0,049	0,006
Sph	Sphingomyelin in serum	NMR	-	-	5,71	0,630	0,622	0,094	0,647	0,629	0,114
TChr	Total Cholesterol in serum	NMR	-	-	0,69	5,189	5,036	1,059	5,601	5,492	1,306
TG RMN	Triglycerides in serum	NMR	-	-	4,17	1,409	1,318	0,424	1,427	1,360	0,483

w3	Omega-3 in serum	NMR	-	-	0,96	0,005	0,005	0,001	0,006	0,005	0,001
